# The P42 peptide and Peptide-based therapies for Huntington’s disease

**DOI:** 10.1186/s13023-016-0405-3

**Published:** 2016-03-17

**Authors:** Cecilia Marelli, Florence Maschat

**Affiliations:** Université de Montpellier, Montpellier F-34095, France; Inserm U1198 MMDN, Montpellier F-34095, France; EPHE, Paris F-75014, France, Montpellier, France; Department of Neurology, Gui de Chauliac University Hospital, Montpellier, France

**Keywords:** Huntington’s disease, Peptide-based therapy, P42

## Abstract

Huntington’s disease (HD) is a progressive neurodegenerative hereditary disease clinically characterised by the presence of involuntary movements, behavioural problems and cognitive decline. The disease-onset is usually between 30 and 50 years of age. HD is a rare disorder affecting approximately 1.3 in 10,000 people in the European Union. It is caused by an expanded CAG repeat in the first exon of the *Huntingtin* (*HTT)* gene, leading to an abnormal form of the Huntingtin protein (Htt) (polyQHtt), containing N-terminus, enlarged polyglutamine strands of variable length that stick together to form aggregates and nuclear inclusions in the damaged brain cells. Treatments currently used for Huntington’s disease are symptomatic and aimed at temporally relieving the symptoms of the disease; although some promising therapies are on study, there is no drug capable of stopping disease progression either in the form of delaying onset or slowing disability progression. The utilization of peptides interacting with polyQ stretches or with Htt protein to prevent misfolding and aggregation of the expanded polyQ protein is a fascinating idea, because of low potential toxicity and ability to target very initial steps in the pathophysiological cascade of the disease, such as aggregation or cleavage process. Indeed, several therapeutic peptides have been developed and were found to significantly slow down the progression of symptoms in experimental models of Huntington’s disease. This review is essentially focusing on the latest development concerning peptide strategy. In particular, we focused on a 23aa peptide P42, which is a part of the Htt protein. It is expected to work principally by preventing the abnormal Htt protein from sticking together, thereby preventing pathological consequences of aggregation and improving the symptoms of the disease. In the meantime, as P42 is part of the Htt protein, some therapeutic properties might be linked to the physiological actions of the peptide itself, considered as a functional domain of the Htt protein.

## Background

Huntington’s disease (HD) is an autosomal-dominant, progressive neurodegenerative disorder clinically characterized by the presence of motor dysfunction (chorea, dystonia, extrapyramidal rigidity and akinesia), cognitive decline (early alteration of executive functions, mental flexibility, and attention; later impairment of language, visuospatial and memory functions), and neuropsychiatric symptoms (depression, apathy, anxiety, obsessive/compulsive behaviours, irritable/aggressive symptoms and sometimes personality changes, and psychosis). Typically, onset of symptoms is in adulthood between 30 and 50 years, but the disorder can manifest at any time between infancy and senescence [1, 2]. HD is a rare disease and the highest prevalence is estimated to be 1.3/10.000 [3].

HD is caused by an expanded CAG repeat in the first exon of the *Huntingtin* (*HTT)* gene [4]; the resulting mutant protein in HD (polyQHtt) contains enlarged polyglutamine repetitions of variable lengths, that stick together and form intranuclear and intracytoplasmic cellular deposits. In initial disease stages, cell loss and reactive gliosis affect predominantly striatal medium spiny neurons, while polyQHtt positive inclusions are found in cortical region; in later disease stages cortical cell loss is found [5–7]. Currently there is no curative treatment for HD; only symptomatic pharmacological and non-pharmacological treatments are available with some benefits mainly for motor and psychiatric HD symptoms [8–11]. Unfortunately, there is no drug capable of stopping disease progression either in the form of delaying onset or slowing symptoms progression [12].

The precise physiopathology of HD is complex and involves many mechanisms that have been described [13, 14]: protein aggregation, alteration of protein degradation through autophagy [15] or ubiquitin-proteasome-system (UPS) [16], enhanced proteolytic cleavages [17], transcriptional deregulation and brain-derived nerve factor (BDNF) alteration [18], mitochondrial abnormalities and defective energy metabolism [19–21], cytoskeletal defects and axonal transport alterations [22, 23], loss of wtHtt normal function [24, 25], non cell-autonomous degeneration [26], and neuro-inflammation [27–29].

Based on these different pathogenic mechanisms, several therapeutic approaches have been proposed to date [30]. Among them, here we focus on peptide-based approaches that target different parts of the polyQHtt, preventing aggregate formation of the polyQHtt proteins, and that were further tested for their ability to rescue abnormal cellular processes induced by the expanded polyQ proteins.

## Peptide-based therapeutic approach in HD and polyglutamine diseases

The utilization of peptides interacting with polyQ stretches or with Htt protein to prevent misfolding and aggregation of the expanded polyQ protein is a promising intervention. A summary of the different peptides developed up to now against HD is presented in Tables 1, 2 and 3.

**Table 1:** Summary of the efficacy of the different peptides against HD

Peptide	Target of the peptide	Model	Population	Way of administration	End point	Method of evaluation	Results
Bivalent Htt-binding peptide (Kazantsev et al., 2002) [31]	PolyQ stretches	Cell culture	COS-1 cells	Co-transfection of hHtt^17aa^-103Q ± bivalent Htt-binding peptide	Aggregation	% of aggregate-positive transfected cells	Delayed aggregate formation: 37.6 % reduction at 48 h; no reduction at 96 h
		Drosophila HD	*ELAV*-Gal4; UAS- 48/108Q	Genetic cross: bivalent Htt-binding peptide *vs* placebo	Survival	Survival rate	Significant increased survival
					Aggregation (CNS)	Immunostaining on L3 larvae	Significant aggregate reduction
			*GMR*-Gal4; UAS- 48/108Q	Genetic cross: bivalent Htt-binding peptide vs placebo	Photoreceptor neurodegeneration	Quantification of the number of rhabdomeres/ommatidium	Significant rescue of eye neurodegeneration
Polyglutamine-binding peptide 1 (QBP1) (Nagai et al., 2000) [32]	Expanded polyQ stretch	Cell culture	COS-7 cells	Co-transfection of 45Q-/57Q-/81Q-YFP ± QBP1-CFP	Aggregation	% of aggregate-positive transfected cells	Significant aggregate reduction, more important with shorter polyQ
(QBP1)_2_ (Nagai et al., 2003) [33]	Expanded polyQ stretch	Drosophila polyQ models	*GMR*-92Q	Genetic cross: *Eyeless*-Gal4; UAS-(QBP1)_2_ or *GMR*-Gal4; UAS-(QBP1)_2_	Photoreceptor neurodegeneration	Phenotypical comparative analysis (adult flies)	Significant suppression of eye degeneration
			*GMR*-Gal4; UAS-MJD^tr^-78Q	Genetic cross: UAS-(QBP1)_2_	Photoreceptor neurodegeneration	Phenotypical comparative analysis (adult flies)	Significant suppression of eye degeneration
			*GMR*-92Q	Genetic cross: *Eyeless*-Gal4; UAS-(QBP1)_2_	Aggregation in the eye imaginal disc	Immunostaining (third instar larvae)	Significant inclusion bodies reduction
			*ELAV*-Gal4; UAS-MJD^tr^-78Q	Genetic cross: UAS-(QBP1)_2_ or UAS-(scrambled)_2_	Survival	Life span (adult flies)	Significant increase in survival (median life span from 5.5 to 52 days)
PTD-QBP1	Expanded polyQ stretch	Cell culture (Popiel et al., 2007) [39]	COS-7 cells	Co-transfection of 81Q-GFP ± Antp-QBP1 provided in the cell medium	Aggregation	% of transfected cells forming inclusion bodies	Significant reduction (from 42 % to 30 %)
			COS-7 cells	Co-transfection of 57Q-GFP ± TAT-QBP1 provided in the cell medium	Cell survival	Quantification of cell death	Significant reduction of cell death (from 11.8 % to 7.4 %)
		Drosophila polyQ model (Popiel et al., 2007) [39]	*ELAV*-Gal4; UAS-MJD^tr^-78Q	Oral administration of Antp-QBP1	Survival	Survival rate (5,10, and 15 days)	Significant increase
					Aggregation in the eye imaginal disc	Immunostaining (third instar larvae)	Significant reduction of inclusion bodies
		Mouse model (Popiel et al., 2009) [40]	R6/2 mice	Long-term continuous intraperitoneal administration of either Antp-QBP1 (2 mg/week) or saline from wk2	Motor performances	Latency to fall with accelerating rotarod (from wk5 to death)	No significant difference
					Body weight	Weight measure (from wk5 to death)	Significant weight increased compared to saline-treated mice from wk5 to 10
					Survival	Life span	No significant difference
				Long-term continuous intraperitoneal administration of either Antp-QBP1 (2 mg/week) or saline from wk2	Aggregation	Brain section immunostaining with anti-htt antibody	No significant difference
ED11 (Aharony et al., 2015) [41]	Inhibitor of caspase-6	Cell culture	PC12 cells	Inducible mHtt- 145Q ± TAT-ED11 provided in the cell medium	Survival	Cell viability and cell death assessment	Significant increased cell viability and decreased cell death
		Mouse model	Full-length hHtt-97Q BACHD	Pre-symptomatic treatment (from wk5); continuous infusion (4 mg/kg/day; subcutaneously implanted mini-pump) of ED11 peptide *vs* vehicle in BACHD mice and of vehicle in wt mice	Body weight (excessive weight)	Weight measure	Attenuation of weight gain
					Motor performances	Latency to fall with accelerating rotarod (monthly from wk9)	Preserved motor performance compared to wt mice.
					Depressive-like behaviour	Immobility evaluation during the forced swim test (FST) (5 months of age)	Prevention of increased immobility
					Basal locomotor activity, exploratory activity, anxiety-related behaviour	Open field test (wk22): total travelled distance; time spent in the centre and number of transitions to the centre	Unchanged basal locomotor activity; lower anxiety levels and improved exploratory behaviour in treated *vs* untreated mice
					Inhibition of caspase-6 activity	Quantification of mHtt^586aa^ fragments (6-month-old mice)	Not evaluable (no detectable mHtt^586aa^ fragments in untreated mice)
					Aggregation	Immunostaining (6-month-old mice)	Not evaluable (no detectable aggregates in untreated mice)
				Post-symptomatic treatment (from w36); continuous infusion (4 mg/kg/day; subcutaneously implanted mini-pump) of ED11 peptide *vs* vehicle in BACHD mice and of vehicle in wt mice	Motor performances	Latency to fall with accelerating rotarod (monthly, wk30 to 44)	Increased motor performance compared to untreated mice
					Depressive-like behaviour	Immobility evaluation during the forced swim test (FST) (11 months of age)	Rescue at the level of wt littermates
					Cognitive deficits	Swimming T-maze test; shifting abilities (time to reach the re-located hidden platform)	Rescue at the level of wt littermates
					Brain atrophy	MRI volumetric measurements (12 months of age)	Not evaluable (no significant atrophy in untreated BACHD mice)

Legend: to characterize Htt fragments we use the general indication Htt^Xaa^-YQ: the length of the fragment is expressed as a number X of amino acids (aa) (superimposed); the length of polyQ expansion is expressed as a number Y of Q.

**Table 2:** Summary of the efficacy of the different intrabodies against HD

Antibody	Target of the peptide	Model	Population	Way of administration	End point	Method of evaluation	Results
C4 intrabody	N17 terminal region	Cell culture (Lecerf et al., 2001) [44]	COS-7; BHK-21; HEK293	Co-transfection: hHtt^17aa^-25/73/103Q-GFP ± C4 intrabody (ratio 5:1)	Aggregation	% of aggregate-positive transfected cells	Reduction up to 86 %
		Organotypic cultures (Murphy and Messer, 2004) [46]	Cortico-striatal slice cultures	Malonate treatment, and transfection with hHtt^17aa^-25/72Q-GFP ± C4 intrabody	Cell survival	% of co-transfected died or dying cells	Rescue to wt level
		Drosophila model (Wolfgang et al., 2005) [45]	ELAV-Gal4; UAS-hHtt^ex1^-20/93Q;	Genetic cross: UAS-C4 intrabody”	Survival	% of survival to adult (eclosion); mean, median, and maximal lifespan	Increased survival to adulthood (from 23 % to 100 %); increased mean adult lifespan by 30-50 %
					Aggregation; quantification of soluble polyQ forms	Immunostaining; detergent-soluble hHtt^ex1^-93Q detection (Western blot)	Slowing of visible aggregate formation. Increased levels of soluble Htt
					Neurodegeneration	Photoreceptors/ommatidium quantification	Slowing of neurodegeneration in photoreceptors cells
		Mouse model (Snyder-Keller et al., 2010) [47]	B6.HD6/1 125Q (hHtt^ex1^-125Q)	C4 intrabody with AAV vector into the striatum; presymptomatic (injection: wk5 to 8 ; killed at wk16 to 32); symptomatic (injection wk 10 to 24; killed 8 to 10 wk later)	Aggregation	Immunostainig: number and size aggregates	Pre-symptomatic and symptomatic effect: aggregate reduction (size > number), more important in pre-symptomatically treated mice
V_L_12.3 intrabody	N17 terminal region	Cell culture (Colby et al., 2004) [48]	HEK293	Co-tranfection: hHtt^ex1^-97Q-GFP + empty vector or V_L_12.3	Aggregation	Immunostaining	50 % reduction of aggregates *vs* empty vector
		Cell culture (Southwell et al., 2008) [49]	HEK293	Co-transfection: hHtt^ex1^-103Q-GFP + empty vector or V_L_12.3	Aggregation	Immunostaining	Dose-dependent aggregate reduction
					Cell survival	% of co-transfected dead cells	Reduced cell toxicity
				Co-transfection: hHtt^ex1^-25/103Q-GFP + V_L_12.3	Quantification of soluble and insoluble hHtt^ex1^	Centrifugation and Immunoblot assay	Significant reduction of insoluble but not of soluble hHtt^ex1^-103Q levels
				Co-transfection: hHtt^ex1^-103Q -SNAP tag ± V_L_12.3	hHtt^ex1^-103Q turnover	Fluorescence intensity of SNAP-tag	No effect on polyQ turnover
		Cortico-striatal brain slice model (Southwell et al., 2008) [49]	Rat brain slices	Co-transfection: YFP as morphometric marker ± hHtt^ex1^-103Q -CFP ± V_L_12.3	Neurodegeneration	Immunostaining: counting of healthy striatal medium spiny neurons (MSNs)	Rescue of neurodegeneration at the level of wt cells
			ST14A striatal precursor cells	Co-transfection: hHtt^ex1^-103Q -GFP ± V_L_12.3	hHtt^ex1^-103Q localisation and turnover	Immunostaining: cytoplasmic/nuclear hHtt^ex1^-103Q ratio	Altered cytoplasmic/nuclear trafficking: significant increase of nuclear Htt
		Mouse model (Southwell et al., 2009) [51]	C57BL/6 (lentiviral HD model)	HD model: Unilateral striatal injection: hHtt^ex1^-103Q -GFP or GFP lentivirus.Treatment: + V_L_12.3- AAV or GFP (4 wk-old mice). Tests 6wks later.	Amphetamine-induced rotation	Ipsilateral rotations tested during 30’ after intraperitoneal amphetamine injection.	Strong reduction of the number of ipsilateral rotations to the levels of GFP lentivirus injected animals
					MSNs loss	DARPP-32 staining	Rescue to the levels of GFP lentivirus injected animals
					Aggregation	Striatal immunostaining with anti-Htt MW8 (detect aggregates only)	Significant aggregate reduction *vs* AAV-GFP injected animals
			YAC128 (Full length-hHtt-128Q)	2-months-old male mice and wt littermates injected bilaterally in the striatum with GFP- or V_L_12.3- AAV	Motor performances	Rotarod latency to fall (monthly from 3 to 7 months of age)	No effect
						Beam-crossing performance (monthly from 3 to 7 months of age)	No effect
						Climbing time (7-month-old mice)	No effect
					Cognitive performances (spatial and cortical learning)	Novel object location and novel object preference tests (7-month-old mice)	No effect in both tests
					Anxiety	Open field test	Non significant amelioration
					Brain atrophy	Ventricular size assessment (7-month-old mice)	No effect
					Body weight	Assessment monthly from 3 to 7 months of age	No effect
			R6/2 (hhtt^ex1^- 144Q)	3-day-old male mice and wt littermates: bilateral injection at the center of each forebrain hemisphere of GFP- or V_L_12.3-AAV	Motor performances	Rotarod latency to fall (weekly from w4 to death)	Reduced latency to fall (wk 10 to 12)
						Beam-crossing performance (weekly from w4 to death)	No rescue: Increased severity of the phenotype (time to cross the beam)
					Brain atrophy	Ventricular size assessment (10-wk-old mice)	No effect
					Body weight	Assessment weekly from 4 wk until death	No effect
					Aggregation	Striatal immunostaining with anti-Htt MW8 (detect aggregates only) and nuclear marker; counting of positive foci (10 week-old mice)	Reduction of the number of neuropil aggregates; no significant reduction of intranuclear aggregates
					Life span	Once ill, twice a day assessment of righting reflex	Aggravation and decrease survival
MW7 intrabody	Poly P region	Cell culture (Khoshnan et al., 2002) [50]	HEK293	Co-transfection: hHtt^ex1^-97Q-GFP and MW7 or empty vector	Aggregated/soluble Htt	Centrifugation, SDS treatment and western blotting	Reduction of both aggregated and soluble polyQHtt
					Cell survival	TUNEL staining	33 % reduction of TUNEL positive cells
		Cell culture (Southwell et al., 2008) [49]	HEK293	Co-transfection: hHtt^ex1^-103Q-GFP + empty vector or MW7	Aggregation	Immunostaining	Aggregate reduction with a threshold-effect
					Cell survival	% of co-transfected dead cells	Reduced cell toxicity
				Co-transfection: hHtt^ex1^-25/103Q-GFP + MW7	Quantification of soluble and insoluble hHtt^ex1^	Centrifugation and Immunoblot essay	Significant reduction of both soluble and insoluble hHtt^ex1^-103Q; no effect on soluble wt hHtt^ex1^-25Q
				Co-transfection: hHtt^ex1^-103Q-SNAP tag ± MW7	hHtt^ex1^-103Q turnover	Fluorescence intensity of SNAP tag	Significant decreased fluorescence (increased hHtt^ex1^-103Q turnover)
		Cortico-striatal brain slice model (Southwell et al., 2008) [49]	Rat brain slices	Co-transfection: YFP ± hHtt^ex1^-103Q -CFP ± MW7	Neurodegeneration	Immunostaining: counting of healthy MSNs	Non-significant reduction of neurodegeneration
			ST14A striatal precursor	Co-transfection: hHtt^ex1^-103Q -GFP ± MW7	hHtt^ex1^-103Q localisation and turnover	Immunostaining: cytoplasmic/nuclear hHtt^ex1^-103Q ratio	No effect
Happ1-Happ3 antibodies	Poly P region	Cell culture (Southwell 2008) [49]	HEK293	Co-transfection: hHtt^ex1^-103Q-GFP + empty vector or Happ1-Happ3	Aggregation	Immunostaining	Dose-dependent aggregate reduction
					Cell survival	% of co-transfected dead cells	Reduced cell toxicity
				Co-transfection: hHtt^ex1^-25/103Q-GFP + Happ1-Happ3	Quantification of soluble and insoluble hHtt^ex1^	Centrifugation and Immunoblot essay	Significant reduction of both soluble and insoluble hHtt^ex1^-103Q; no effect on soluble wt hHtt^ex1^-25Q
				Co-transfection: hHtt^ex1^-103Q-SNAP tag ± Happ1-Happ3	hHtt^ex1^-103Q turnover	Fluorescence intensity of SNAP tag	Significant decreased fluorescence (increased hHtt^ex1^-103Q turnover)
		Cortico-striatal brain slice model (Southwell et al., 2008) [49]	Rat brain slices	Co-transfection: YFP as morphometric marker ± hHtt^ex1^-103Q -CFP ± Happ1-Happ3	Neurodegeneration	Immunostaining: counting of MSNs	Significant reduction of neurodegeneration
			ST14A striatal precursor	Co-transfection: hHtt^ex1^-103Q -GFP ± Happ1-Happ3	hHtt^ex1^-103Q localisation and turnover	Immunostaining: cytoplasmic/nuclear hHtt^ex1^-103Q ratio	No effect
		Mouse model (Southwell et al., 2009) [51]	C57BL/6 (lentiviral HD model)	HD model: Unilateral striatal injection: hHtt^ex1^-103Q -GFP or GFP lentivirus.Treatment: + GFP- or Happ1- AAV (4 wk-old mice). Tests 6wks later.	Amphetamine-induced rotation,	Ipsilateral rotations tested during 30’ after intraperitoneal amphetamine injection.	Strong reduction of the number of ipsilateral rotations to the levels of GFP lentivirus injected animals
					MSNs loss	DARPP-32 staining	Rescue to the levels of GFP lentivirus injected animals
					Aggregation	Striatal immunostaining with anti-Htt MW8 (detect aggregates only) and nuclear marker; counting of positive foci.	Significant aggregate reduction *vs* AAV-GFP injected animals
			YAC128 (Full length-hHtt-128Q)	2-months-old male mice and wt littermates injected bilaterally in the striatum with GFP- or Happ1- AAV	Motor performances	Rotarod latency to fall (monthly from 3 to 7 months of age)	Improvement in 3-, 4-, and 7 –month-old mice
						Beam-crossing performance (monthly from 3 to 7 months of age)	Partial improvement
						Climbing (7-month-old mice)	Increased climbing time to the level of wt littermates
					Cognitive performances (spatial and cortical learning)	Novel object location and novel object preference tests (7-month-old mice)	Significant amelioration of spatial and cortical learning
					Anxiety	Open field test	Rescue to the level of wt littermates
					Brain atrophy	Ventricular size assessment (7-month-old mice)	Reduction of ventricular size
					Body weight	Assessment monthly from 3 to 7 months of age	No effect
			R6/2 (hhtt^ex1^- 144Q)	3-day-old male mice and wt littermates: bilateral injection at the center of each forebrain hemisphere of GFP- or Happ-AAV	Motor performances	Rotarod latency to fall (weekly from 4 wk until death)	Amelioration (between w9 and 12 of age) *vs* GFP-AVV injected animals.
						Beam-crossing performance (weekly from 4 wk until death)	Reduction of the time to cross the 12 mm beam in 10- and 11-week-old mice, and the 6 mm beam between 9 and 11 weeks of age
					Brain atrophy	Ventricular size assessment (10-wk-old mice)	Reduction of ventricular size to the level of wt littermates
					Body weight	Assessment weekly from 4 wk until death	No effect
					Aggregation	Striatal immunostaining with anti-Htt MW8 (detect aggregates only) and nuclear marker; counting of positive foci (10 week-old mice).	Reduction of the number of both neuropil and intranuclear aggregates.
					Life span	Once ill, twice a day assessment of righting reflex	No effect
			N171-82Q: hHtt^171aa^-82Q	Four-week old male mice and wt littermates: bilateral striatal injection of GFP- or Happ1-AAV	Motor performances	Latency to fall with accelerating rotarod (every 2 wks from wk6 until death)	Significant improvement from wk 20 to 40 at the level of wt mice
						Beam-crossing performance (every 2 wks from wk6 until death)	Significant improvement with reduction of time to cross the three beams *vs* GFP-AAV treated and wt mice.
						Clasping (22-week-old mice)	Attenuation of clasping behavior
					Body weight	Assessment every 2 wks from wk6 until death	Increased weight *vs* GFP-AAV treated but not to the level of wt littermates (from wk 22)
					Life span	Once ill, twice a day assessment of righting reflex	33 % increase of maximum life-span (from 30 to 40 wk) *vs* GFP-AAV treated mice.
			BACHD: Full-length- hHtt-97Q-	2-months-old male mice and wt littermates: bilateral striatal injection of GFP- or Happ1-AAV	Motor performances	Rotarod latency to fall (monthly from month 3 to 6)	Increased latency to fall in 5- and 6- month-old mice
						Beam-crossing performances (monthly from month 3 to 6)	Decrease time to cross the beams at 5 and 6 months (28 mm beam) and at month 6 (6 mm beam)
						Climbing time (6-month-old mice)	Increased climbing time *vs* GFP treatment
					Cognitive performances (spatial and cortical learning)	Novel object location and novel object preference tests (6-month-old mice)	No effect
					Anxiety	Open field test	Significant effect *vs* GFP-AAV treated mice
					Brain atrophy	Ventricular size assessment (6-month-old mice)	Reduction of ventricular size
					Body weight	Assessment monthly (from 3 to 6 months of age)	No effect
mEM48 intrabody (Wang et al., 2008) [52]	VA residues after the polyP region	Cell culture	HEK293	Co-transfection: hHtt^208aa^ 23/130Q ± EM48	Cell survival	% of co-transfected dead cells	Improved cell viability
			Rat cortical neurons	Co-transfection: hHtt^208aa^ 23/130Q ± EM48	Neuritic disruption and pyknotic nuclei	Neuronal morphology	Significant reduction of transfected neurons with disrupted neurites or fragmented nuclei
			PC12 cells	Transfection of hHtt^208aa^ 23/130Q ± AAV-EM48	Neuropil aggregates	Immunostaining	Significant reduction of neuropil aggregates
		Mouse model	R6/2 (hhtt^ex1^- 144Q)	Intrastriatal injection of helper dependent AAV EM48 (7-wk-old mice)	Neuropil aggregates	Immunostaining (4 wk after injection)	Significant less neuropil aggregates *vs* non injected region; no effect on intranuclear inclusion
			N171-82Q	Bilateral striatal injection of helper dependent AAV EM48 (10-wk-old mice)	Neuropil aggregates	Immunostaining (6 wk after injection)	Significant less neuropil aggregates *vs* non injected region; no effect on intranuclear inclusions
					Motor performances	Stride length (8-wk post injection)	Improvement
						Rotarod latency to fall (8-wk post injection)	Significant improvement
					Body weight		No effect
					Survival		No effect
Monoclonal antibodies 1C2 (Heiser et al., 2000 [43]; Trottier et al., 1995) [42]	PolyQ chain (soluble)	Cell culture	COS-1	Co-transfection: hHtt^ex1^-51Q ± 1C2	Aggregation	Filter retardation assay	Up to 85 % reduction in aggregates

Legend: To characterize Htt fragments we use the general indication Htt^Xaa^-YQ: the length of the fragment is expressed as a number X of amino acids (aa) (superimposed); the length of polyQ expansion is expressed as a number Y of Q.

**Table 3:** Summary of the efficacy of P42 in cellular, *Drosophila*, and mouse R6/2 HD models

Peptide	Model	Population	Way of administration	End point	Method of evaluation	Results
P42 (Arribat et al., 2013) [55]	Cell culture	HeLa cells (hHtt^171aa^-136Q)	Co-transfection: polyQHtt + P42 or empty vector	Htt aggregation	Immunostaining; filter retardation assays	Rescue = 80 %
P42TAT (Arribat et al., 2014) [61]	Cell culture	HeLa cells (hHtt^171aa^-136Q)	Co-transfection: polyQHtt + P42TAT or empty vector	Htt aggregation	Immunostaining; filter retardation assays	Rescue = 80 %
			P42TAT-TAMRA provided in culture cell medium	Htt aggregation	Immunostaining; filter retardation assays	Rescue = 90 % (P42TAT concentration dependent)
P42 (Arribat et al., 2013) [55]	HD Drosophila	*MS-1096*-Gal4; UAS-HA-hHtt^171aa^-138Q	Genetic cross: UAS-P42 *vs* UAS-LacZ (neutral control)	Htt aggregation	Immunostaining; filter retardation assays (L3 larval salivary glands)	Rescue = 80 %
		*GMR*-Gal4; UAS- hHtt^ex1^-93Q	Genetic cross: UAS-P42 *vs* UAS- GFP (neutral control)	Eye toxicity	Phenotypical comparative analysis (eyes of adult flies)	Rescue = 100 %
		*OK6*-Gal4/UAS-NPY-GFP; UAS-hHtt^548aa^-128Q	Genetic cross: UAS-P42 *vs* UAS-LacZ (neutral control)	Larval locomotion	Locomotion (mm/min)	Rescue close to 100 %
		*OK6*-Gal4/UAS-NPY-GFP; UAS-hHtt^548aa^-128Q	Genetic cross: UAS-P42 *vs* UAS-LacZ (neutral control)	Axonal transport	Immunostaining and live imaging to quantify different parameters of Neuropeptide Y vesicles trafficking in larval motoneurons.	Recovery of the different parameters: Number of vesicles: 28 %; % of pausing: 21 %; velocity: 31 %
		*ELAV*-Gal4; UAS-hHtt^548aa^-128Q	Genetic cross: UAS-P42 *vs* UAS-LacZ (neutral control)	Adult survival	Mean, median, and maximal survival (days)	Increased median survival (day 18 to 26); no effect on mean and maximal survival
P42TAT (Arribat et al., 2014) [61]	R6/2 mice	hHtt^ex1^-140Q	Transmucosal daily administration of P42TAT with Aonys® water-in-oil microemulsion (600 μg/ml/kg) *vs* empty microemulsion at pre-symptomatic (wk2 to wk11) R6/2 and Wt mice.	Motor performance	Latency to fall from accelerating rotarod (weeks 6, 8, and 10)	Significant amelioration compared to placebo-treated R6/2 mice
				Clasping test	Frequency and duration of the foot-clasping posture (twice a week at wk 7, 9, and 11)	Complete rescue *vs* placebo-treated R6/2 and to wt mice
				Weight loss	Weight measure between wk8 and wk10	Significant reversion of body weight loss curve *vs* placebo-treated mice
				Intranuclear brain aggregates; astrogliosis	Immunostaining: number and size of cortical and striatal intranuclear aggregates; cortical and striatal astrocyte number	Significant 50 % reduction of cortical and striatal aggregates; non significant reduction of the astrogliosis
				Cerebral atrophy	Lateral ventricle enlargement	Rescue = 30 %

Legend: to characterize Htt fragments we use the general indication Htt^Xaa^-YQ: the length of the fragment is expressed as a number X of amino acids (aa) (superimposed); the length of polyQ expansion is expressed as a number Y of Q.

One of the first proposed peptide was a synthetic bivalent Htt-binding peptide containing two stretches of short polyQ regions (25Q), separated by an alpha-helix structure; it co-localized with aggregate of polyQHtt protein and interacted with the polyQHtt. This peptide was able to reduce and delay aggregate formation in a cellular HD model (COS-1 hHtt^17aa^-103Q); moreover it increased survival, reduced eye aggregate formation and degeneration, and inhibited brain aggregate formation in Drosophila HD models [31].

Polyglutamine binding peptide 1 (QBP1) is a 11 aa synthetic peptide identified by a combinatorial screening approach for its specific binding affinity to abnormal expanded polyQ stretch [32]. QBP1 was able to co-localize with and to reduce aggregate formation in cultured cells; in Drosophila HD model it increased survival and decreased eye degeneration and aggregate formation [33]. To allow its efficient cellular delivery, QBP1 was conjugated to short peptides belonging to the group of the peptide transduction domains (PTD) or cell penetrating peptides (CPP), like the Penetratin part of Antennapedia (Antp) [34] or of the HIV TAT-derived protein, allowing the access to the cytoplasm and the nucleus after their internalization by living cells [35–38]. This technique overcame the problems of intestinal membrane passage and increased bioavailability after administration of the modified peptide in Drosophila food: oral administration of Antp-QBP1 to polyQ-Macado Joseph Disease (MJD) flies, a Drosophila model of the polyQ-induced spinocerebellar ataxia 3 disease (SCA3), remarkably delayed premature adult flies death; in addition, polyQ-MJD flies administered with Antp-QBP1 had significantly fewer polyQ aggregates in the eye imaginal disc of third instar larvae, compared to the control flies [39]. The therapeutic effect of Antp-QBP1 administration was also tested on a R6/2 mouse model of the polyQ disease [40]: intraperitoneal injection of Antp-QBP1 resulted in a slight improvement of the weight loss, but did not improve the other phenotypes such as motor dysfunction and premature death; no significant decrease of polyQ inclusion body formation could be detected. After intra-cerebroventricular and intra-striatal injection of Antp-QBP1 or TAT-QBP1 peptides into wild type (wt) C57BL/6 mouse, PTD-QBP1 showed limited diffusion into the brain, restricted to a few cell layers around the ventricles with however a more efficient diffusion for Antp-QBP1. After either intra-peritoneal or intracarotid arterial injection, no detectable levels of PTD-QBP1 were found into the brain. The authors suggested that lack of efficacy was due either to low targeting of PTD-QBP1 into the brain or to a too severe phenotype in the R6/2 mouse model [40].

More recently, a caspase-6 inhibiting peptide, targeting the cleavage of the polyQHtt protein, a key step in HD pathogenesis, was proposed and tested on the full-length 97Q-mHtt transgenic BACHD mouse model [41]. This 24aa peptide, called ED11, was designed on the basis of the caspase-6 cleavage site in N-terminal part of Htt and was able to inhibit caspase-6 activity by competing with the caspase-6 active site on Htt; to enable cell penetration ability, the HIV TAT-derived peptide was used. The authors accurately showed the selective caspase 6 interference effect, with an only minor additional effect on caspase 1 and 10 cleavage sites. Sub-cutaneous continuous administration of ED11 with a minipump at a pre-symptomatic stage showed restoration of body weight, preserved motor performances, less depressive behaviour and improved cognitive deficits. At a post-symptomatic stage, ED11 administration showed amelioration on motor performances, cognition, and depression. Unfortunately, in this full-length hHtt-97Q transgenic BACHD mouse model, neither aggregation was detectable, nor significant atrophy was found, making impossible the evaluation of the efficacy of the ED11 peptide on these features. Importantly no toxicity in cell or in mouse after prolonged administration was found [41].

Monoclonal antibodies were also generated, such as 1C2 [42], able to specifically recognize the conformation of an elongated polyQ form in soluble proteins, but not the CAG sequence in insoluble aggregates of polyQ proteins, suggesting that 1C2 reduced aggregation, probably by stabilizing the polyQ protein in a native, soluble form and preventing aggregation-prone conformational changes [43].

Finally, several intracellular antibodies, known as intrabodies, binding to different part of the N-terminal Htt have also been identified to date. The intrabodies identified so far recognize a region in Htt other than the polyQ stretch itself and are therefore potentially specific for Htt protein, although cross-reactivity with other proteins cannot be excluded. Some intrabodies, like C4 [44–47] and V_L_12.3 [48] bind to the first 17aa of Htt (N17 region): they act by preventing aggregation and forming soluble complexes with the Htt-N-terminal part, which subsequently may undergo normal protein turnover; the levels of soluble wt and polyQHtt were therefore reported as increased [45] or unchanged [49] (Table 2). Other intrabodies (HAPP1, HAPP3, and MW7) [49] [50, 51] bind to the Proline Reach Region (PRR) domain: they act mainly by enhancing the degradation of the mutant protein which reduces soluble polyQHtt levels [49] (Table 2). Finally, another intrabody (mEM48), directed to the Valine/Alanine amino acid residues after the PRR tract, might alter the conformation of mutant Htt, leading to its degradation *via* the ubiquitin-proteasome system [52]. The expression of these intrabodies was shown to be efficient in suppressing Htt aggregation and neurodegeneration when tested in a *Drosophila* model, through genetic expression in transgenic flies, [45] and in mouse models of HD, through conjugation with a viral vector and after an intra-striatal injection [47, 51, 52] (Table 2). The use of intrabodies is therefore an attractive therapeutic approach with regard to their high binding affinity to the disease-causing proteins and their potential specificity for HD. Moreover some intrabodies are very small, therefore reducing problems of immunogenicity and allowing the passage in nuclear pore; this could be particular important considering the toxic role of some cleaved N-terminal Htt fragments and their nuclear localisation [53]. However, major concerns about the therapeutic utilisation of intrabodies are the way of administrations and the possible unfavourable side effects due to cross-reactivity with the wild type Htt or to the fact that they are non-physiological entities.

Globally antibody- or peptide-based therapies seem to be very efficacious in ameliorating biological and clinical features of HD in different models; major drawbacks for their *in vivo* use are their rapid degradation by proteases and their poor blood–brain barrier and cellular membranes permeability, leading to an important difficulty in targeting central nervous system (CNS) neurons.

## Identification of the P42 peptide and efficacy on cellular and Drosophila HD models

We have recently identified a new domain of Htt that is also acting on aggregation. We first observed that the 548 aa N-terminal part of normal human Htt (hHtt) or the 620 aa N-terminal part of *Drosophila* Htt homolog (dHtt) were sufficient to prevent polyQHtt aggregation in both HeLa cell and fly HD models [54]. Therefore, we searched for N-terminal region peptides sharing homologies between the human and the fly Htt protein, and playing a protective role on polyQHtt-induced phenotypes [55]. We first sequentially screened peptides designed from the hHtt protein, and identified a 23 aa peptide (P42) that shared homologies with its *Drosophila* counterpart.

**Fig. 1:**
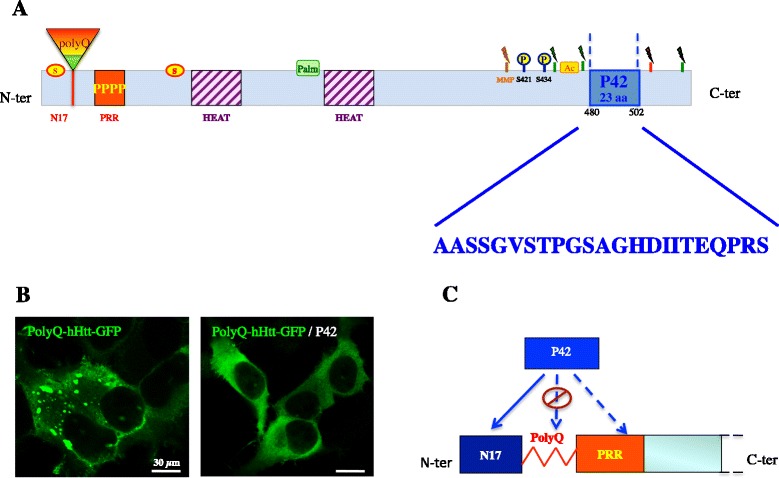
The P42 peptide. A- Location of P42 peptide within the 548 aa N-terminal part of human Huntingtin (hHtt) protein. In the schematic diagram the different domains are indicated: Polyglutamine tract (PolyQ), N17 and Proline rich (PRR) domains covering exon 1, as well as the HEAT repeats; the sites of cleavage by caspase (in red), calpain (in green) or metallomatrixprotease (MMP); posttranslational modifications, such as sumoylation (S), palmitoylation (palm), acetylation (Ac) and some of the phosphorylation (P) sites (modified from [76]). The amino acid sequence of P42 is shown (in blue). B- Cultured HeLa cells co-transfected with polyQ-hHtt-GFP presenting cytoplasmic aggregates (in green); co-transfection with polyQ-hHtt-GFP and P42 prevents aggregate formation [55]. C- Possible mechanism of action of P42 is an interaction with the Htt protein at the N17 or at the Proline Reach region (PRR); interaction with the polyQ domain was excluded on the basis of lack of efficacy in other polyQ-induced disease models [55]. Co-immunoprecipitation and BiFC experiments confirmed a direct interaction of P42 with N17.

The P42 peptide (“AASSGVSTPGSAGHDIITEQPRS”) is derived from the 480–502 aa region of the endogenous hHtt and lies within a region rich in proteolytic sites that play a critical role in the pathogenesis process (Fig. 1a) [56–58]. When the P42 sequence was included in an expressing vector and provided by co-transfection to polyQHtt HeLa cells, it was able of inhibiting polyQHtt aggregation, as efficiently as human longer peptides covering this domain: Htt-548aa or P4-166aa [55] (Fig. 1b). P42 was subsequently tested in *Drosophila* models of HD. To this end, flies expressing polyQHtt were crossed with transgenic flies expressing P42. P42 was found to reduce polyQHtt cytoplasmic aggregates in larval salivary glands and motoneurons, to ameliorate larval locomotion, and to prevent the polyQHtt-induced alteration of vesicular trafficking along the axons of larval motoneurons. P42 also prevented polyQ-induced eye neurodegeneration, characterized by eye depigmentation and abnormal ommatidial arrays; P42 expression in transgenic polyQ flies does not increase survival, although it ameliorates the median of the survival [55] (Table 3).

## Modifications of P42 and efficacy on cellular and mouse HD models

### Conjugation of P42 with a protein transduction domain

To overcome the problems of cell membrane penetration and brain delivery, we conjugated P42 to a cell penetrating peptide (CPP), as already developed for QBP1 [39, 40]. However, instead of using Antennapedia, we used a 11-aa peptide (YGRKKRRQRRR) part of the TAT protein, derived from the HIV; the same CPP was subsequently conjugated to the caspase −6 inhibiting ED11 peptide, and found efficient after intra-peritoneal administration [41]. In HeLa cells expressing polyQHtt, the fusion peptide P42-TAT supplemented in cell culture medium was able to penetrate cells and prevent aggregate formation [55] (Table 3); increasing concentrations of P42-TAT synthetic peptide (from 0.1 μM to 20 μM) drove a clear dose**–**response effect with a complete inhibition of aggregation in presence of 10 μM peptide (Table 3). Note that even a 20-fold excess of this protective dose only produced 25 % mortality, as assessed by the MTT (3–(4,5-dimethylthiazol-2-yl)-2,5-diphenyltetrazolium bromide) colorimetric test, while the IC50 value could not be measured, suggesting a low toxicity of the peptide on cell survival. In case of other small molecules [43], intrabodies, [49, 50, 52], or peptides [41], IC50 value was rather calculated in presence of polyQHtt protein to measure their efficacy in blocking aggregation and cell toxicity.

## Insertion of P42TAT into water-in-oil microemulsion, transmucosal administration and efficacy on mouse model

Although the conjugation to TAT overcame the problem of cell membrane penetration, the issues of peptide degradation, delivery to the central nervous system, and non-invasive administration were still unresolved. In order to optimize the pharmacokinetic characteristics of P42 (serum half life and distribution profile) and to provide a non-invasive route for repetitive delivery of this fusion peptide, we used a novel water-in-oil microemulsion drug delivery vector named Aonys®, developed by Medesis Pharma (France) [59, 60]. Aonys® provides a transmucosal (buccal/rectal) route for drug administration which enhances CNS penetration [61]. This technology was also used for efficient CNS targeting after systemic delivery of lithium in a YAC128 mouse model of HD [62] and of small interfering RNA (siRNA) in a mouse model of prion disease [63]. The studies on P42 diffusivity showed that 3 h after intra-cerebroventricular injections of P42TAT in wild-type C57BL/6 J mice, a tagged form of the peptide (TAMRA-P42TAT) could widely diffuse and be found in different neuronal populations (neurons and astrocytes) and in different cellular compartments (nucleus and cytoplasm), in both the cortex and striatum. The same results were obtained after transmucosal P42TAT water-in-oil microemulsion administration. These data showed that P42TAT has the ability to diffuse into the brain in the different cell layers, including the striatum and that P42-TAT is able to reach the brain when administrated orally *via* Aonys® microemulsion.

The protective effect of Aonys® -formulated fusion peptide was tested in the R6/2 HD mouse model: knowing that symptoms appear at week 6 in these mice, daily transmucosal administration of P42TAT was performed at pre-symptomatic stage (from week 2 to week 11), and was able to improve the motor performances and to reduce polyQ aggregation in the brain, weigh loss, and brain atrophy; the administration of P42 was proved to be efficacious in the R6/2 HD mouse leading to an improvement of at least 30 % recovery, according to the phenotype analysed [61] (Table 3).

## P42 mechanism of action on aggregation process

Protein aggregation is a multistep process requiring an initial event, called nucleation, involving the N17 domain; during this nucleation step, polyQHtt adopts a structure able to associate with itself, which leads to an enhanced local concentration and oligomerization of the mutant hHtt; because of the presence of abnormal expanded polyQ stretches, these oligomers will further form parallel ß sheets, seeding of the aggregation process [64]. The presence of N17 domain was shown to accelerate the aggregation process: intrabodies or proteins targeting the N17 domain are able to suppress aggregation and associated toxicity by inhibiting the initial nucleation step [44, 45, 47, 65–67]. Intrabodies against the PRR domain are also able to reduce polyQHtt aggregation and toxicity, mainly increasing turnover rate of mutant Htt [49, 51].

**Fig. 2:**
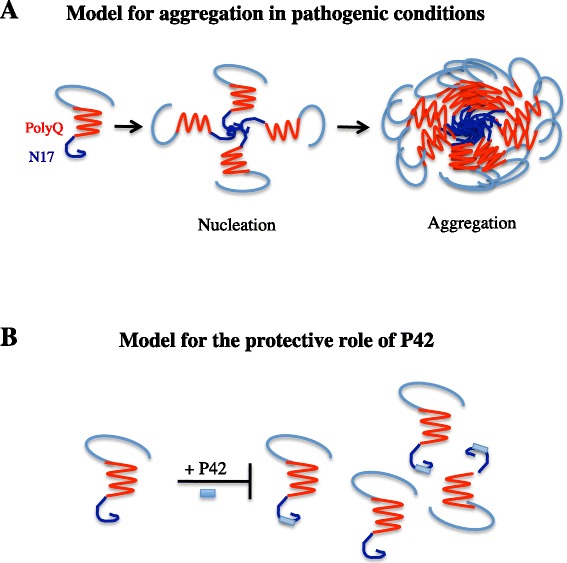
Model of action of P42. A- In pathologic conditions, cleavage of mutant polyQHtt is increased, leading to short N-terminal fragments mostly lacking P42. N17 domains self interact, bringing together polyQHtt proteins (nucleation step). Oligomers will further form parallel ß sheets, thereby enhancing the aggregation process [64]. B- Our model is that exogenous addition of P42 allows a protective effect of polyQHtt-induced defects by directly interacting with the N17 domain of the N-terminal part of polyQHtt, therefore preventing nucleation, and consequently oligomerisation and aggregation processes.

P42 showed to be able to improve phenotype in HD model expressing an expanded polyQ exon1 containing the N17, the PRR, and the polyQ domains, all playing a role in polyQHtt aggregation, suggesting a possible direct interaction between P42 and one of these N-terminal domains. Interestingly, genetic data from Drosophila models highlighted that P42 is not able to counteract eye degeneration in other polyQ-induced diseases different from HD, indicating that P42 mechanism of action is specific for Htt protein and therefore excluding an interaction with the polyQ domain [55]. Indeed, preliminary data including co-IP experiments and Bimolecular Fluorescent Complementation (BiFC) experiments suggest that P42 directly interacts with the N17 domain, therefore confirming the genetic data. This leads us to propose a model in which the addition of exogenous P42 interferes on aggregation process by blocking the initial step of nucleation, through its direct interaction with the N17 domain (Fig. 2).

However, the P42 mechanism of action could be more complex and polymorph: as a part of the Htt protein, it could be a functional protein domain and we cannot exclude a therapeutic effect linked to its normal physiological properties. Interestingly, P42 localised to the Tubulin network *in vivo* in Drosophila cells [54], and recently we confirmed that P42 binds microtubules (unpublished results). As Htt protein is involved in axonal trafficking [68] and P42 ameliorates the vesicular transport along the axons [55], P42 could therefore have a beneficial role modulating axonal transport, independently of its direct effect on aggregation.

## Conclusion

Promising therapeutics for HD are under evaluation, notably nucleotide-based gene silencing methods. Both adeno-associated virus (AAV2) vector expressing *HTT*-silencing micro RNA (miRNA) [69] and intra-ventricular delivered antisense oligonucleotides (ASO) [70] were able to reduce the level of Htt mRNA and protein and to determine an amelioration on motor and behavioural features in different mouse (YAC128, BACHD, R6/2), and non human primate models of HD. Importantly, these studies showed an amelioration on already symptomatic mouse models, suggesting the reversibility of some features of the disease, although earlier treatments produce quicker and more robust reversal of disease; moreover, these studies showed that ASO injection determined a long-lasting transient suppression of Htt protein level, overcoming the period of ASO infusion, and that the amelioration of the disease in turns was evident for an extended period of time, exceeding the period of Htt suppression [70]. The existence of a phase I trial with ASO in humans in Amyotrophic Lateral Sclerosis [71] showing no acute toxicity and the possibility to measure mHtt level in CSF [72] in humans opened the way to a phase I study with intrathecal ASO in symptomatic HD patients. However some important issues are still opened such as selectivity toward mHtt, consequences of a possible concomitant lowering of normal Htt, sufficient targeting and diffusion in the brain after CSF infusion [30].

Whereas peptides and ASO are targeting early events, other new treatments are conceived with symptomatic-only effect or targeting later pathophysiological events and downwards interactors in the disease cascade, such as BDNF, the sirtuin system, or innate inflammation [30, 73].

Peptide-based drugs have been recently introduced in pipeline development and approved for therapy, notably to treat gastrointestinal disorders, hematological cancer, respiratory distress syndrome, Cushing syndrome, and anaemia in chronic kidney disease [74]. Physiological peptides can be biologically active and can be essential component of cell signalling pathways, immune system, hormonal systems, enzymatic systems and other important systems in the body. In the case of neurodegenerative diseases, various routes of delivery can be used: they could be injected to patients and newer methods like intranasal delivery [75], but also oral/rectal mucosal administrations, are presently under investigation [61, 63].

Compared to other synthetic small molecule drugs, physiological peptides might offer lower toxicity, higher specificity, and fewer side effects [74]. Although most of the peptides developed against HD are artificially–conceived peptides, P42 has the advantage to be derived from a sequence physiologically present in the Htt protein; this could be important to limit possible adverse events linked to immunogenicity.

As discussed in this review, peptide-based strategies could be potentially very efficacious in HD treatment because of their ability to target very initial steps in the pathophysiological cascade of the disease, such as aggregation or cleavage process. The efficacy of the different peptides studied until now was demonstrated in different models and on different phenotypes, including polyQHtt aggregation, eye degeneration, survival, larval motility, and axonal transport in the Drosophila HD model, and motor phenotype, weight loss, cognitive performances, cerebral polyQ aggregates, and cerebral atrophy in HD mouse model (Tables 1, 2 and 3). R6/2 mice are one of the models most frequently used to test peptide efficacy since it recapitulates many features of the disease found in humans with HD, such as intranuclear inclusion, weigh loss, motor and cognitive impairment, and brain atrophy. Remarkable results were obtained with peptide-based therapies in the R6/2 model concerning motor performances, body weight, aggregate formation, and brain atrophy; a prolongation of the lifespan was however only observed in the N171-82Q mouse model (Tables 1, 2 and 3). Other HD mouse model, such as full-length hHtt-97Q BACHD or YAC128 were most frequently used to test cognitive performances or anxiety, but in these models, the correct evaluation of the effect on Htt nuclear inclusions, brain atrophy and weight was complicated by the presence of milder or different (such as weight gain instead of weight loss) phenotypes in these less aggressive HD models (Tables 1 and 2). None of the peptide developed until now has been tested in knock-in HD mouse -models.

Up to now, peptide stability and ability to target the central nervous system were major difficulties in the development of peptide at therapeutic ends. These problems were overcome during the development of P42 by conjugation with the TAT peptide transduction domain and using the water-in-oil microemulsion system developed by Aonys® technology. These strategies allowed systemic non-invasive delivery associated with increased peptide stability and efficacious targeting of the central nervous system. Indeed, the association of P42 to TAT and microemulsions allowed reducing the amount of peripheral-administered peptide required to get sufficient peptide level in the central nervous system; non-invasive transmucosal oral administration could be an important issue in the context of a potential chronic administration to pre-symptomatic individuals to ensure patient compliance; finally, peptide stability could be further ameliorated and P42 administration can be potentially associated to other treatments to improve HD prognosis.

P42 could be particularly interesting because of its double role in targeting aggregation and in favouring some physiological function of normal Htt, as it is naturally part of the Htt protein. Although P42 seems efficacious mainly at a pre-symptomatic stage we also found some effect at post-symptomatic stage in R6/2 mice, beginning treatments at week 7 when symptoms already started. Notably, an efficacy on motor performances and brain atrophy was observed after P42 administration to already symptomatic R6/2 mice. Further investigations suggested a beneficial effect of P42 on BDNF level, synaptic plasticity and neuronal activity, behavioural and cognitive aspects of the disease (unpublished results).

Therefore these results suggest that P42 offers a particular therapeutic potential not only by counteracting the effect of the mutant mHtt protein, but also by enhancing the physiological performances of the normal Htt protein. On the basis of these data P42 has recently obtained the designation as orphan medication for HD by the European Medicines Agency (EMA).

## Abbreviations

AAV2: adeno-associated virus
Antp: Antennapedia
ASO: antisense oligonucleotide
BDNF: brain-derived nerve factor
BiFC: bimolecular fluorescent complementation
CNS: central nervous system
CPP: cell penetrating peptides
dHtt: *Drosophila*Huntingtin protein
EMA: European medicines agency
HD: Huntington’s disease
hHtt: human Huntingtin protein
*HTT*: *huntingtin* human gene
Htt: Huntingtin protein
mHtt: mouse Huntingtin protein
miRNA: micro RNA
MJD: Machado-Joseph disease
MSN: striatal medium spiny neurons
MTT: 3–(4,5-dimethylthiazol-2-yl)-2,5-diphenyltetrazolium bromide
polyQ: abnormal expanded polyglutamine strand
polyQHtt: mutant Htt protein with expanded polyQ
PRR: proline reach region domain
PTD: peptide transduction domains
SCA3: spinocerebellar ataxia 3
siRNA: small interfering RNA
wt: wild type
